# A Comparative Analysis of Cultural Competence in Beginning and Graduating Nursing Students

**DOI:** 10.1155/2013/929764

**Published:** 2013-05-23

**Authors:** Helen Reyes, Lance Hadley, Deborah Davenport

**Affiliations:** Department of Nursing, West Texas A&M University, P.O. Box 60969, Canyon, TX 79016, USA

## Abstract

The ethnic proportions of the population in the United States are rapidly changing, with the nation's minority population at approximately 101 million. This is also true for the West Texas region, where locally in a city with 183,000 residents, 43 different languages are spoken suggesting that cultural education needs to be included in nursing program curricula. Therefore, a study was conducted during a period of curriculum revision to determine if the current nursing curriculum at a public university offers enough education and experience for graduating nurses to care for such a diverse population by comparing their perceptions of cultural competence with beginning sophomore nursing students' perceptions. Participants were asked to complete the Cultural Competence Assessment (CCA) tool in order to evaluate perceptions of cultural competence. Upon analysis of the data, perceptions of cultural competence among graduating nursing students were significantly higher (*P* = .002) than the perceptions of cultural competence among beginning nursing students. These results support that nursing students perceive that they have become culturally competent during their nursing education, leading to implications of the need for continued education relating to this concept, beginning with the first course and continuing throughout the nursing curriculum.

## 1. Introduction

Cultural diversity is an issue that faces all health-care workers today. The immigrant population in the United States is increasing, which suggests that education is needed in transcultural nursing to allow nurses to provide culturally competent care. The nation's minority population has reached approximately 101 million, and it is estimated that one in three United States residents is a person belonging to a minority group [1–3]. It is expected by the year 2050 that the Black, Hispanic, and Asian ethnic group percentages within the population will rise dramatically, and the Caucasian population will drop significantly [4], with the USCB (2013) projecting that the United States will become a majority-minority nation, no single group making up the majority, by 2043. Yet, according to the Department of Health and Human Services (DHHS) Office of Minority Health [5], it is estimated that only 10% of registered nurses in the United States claim minority group status. The lack of diversity in the nursing profession mandates a need for cultural competence education in order to provide culturally competent care to an ethnically diverse patient population that is increasing [6, 7].

 Currently, there are no education requirements from the Texas Board of Nursing for transcultural nursing [8]. Cultural competence education concepts remain an option for professional nursing education programs. The American Association of Colleges of Nursing (AACN) believes that diversity and equality of opportunity are core values of all educational systems, and one goal is to create a community of culturally competent scholars, including faculty, students, staff, and practitioners [9]. Decreasing inequities in the health-care system will require culturally competent nurses and other health care providers to meet the needs of disenfranchised groups [10]. The Joint Commission on Accreditation of Health-care Organizations [11] emphasizes the importance of cultural competence in terms of safety and outcomes and believes that it is now essential for nurses to examine their practices related to cultural elements.

 The challenge is growing for professional nurses to care for a culturally diverse population. The American Nurses Association (ANA) believes that nurses and health care providers have a responsibility to provide an environment that recognizes differences and is free from discrimination, including discrimination based on racial and ethnic differences [12]. It is difficult to remain aware of the myriad of cultural groups that reside in the United States. However, health-care professionals should be aware of the dominant cultures within their local area. For example, there are approximately 43 different languages spoken in a city with a population of 183,021 people, which is located in the southwestern United States. Increasing awareness of these different groups will allow nurses to gain additional insight regarding the care of these individuals. 

 According to the ANA, nurses should provide care with respect for human dignity as well as considering the uniqueness of the individual client [12]. Unrealized or unacknowledged biases may prevent nurses from providing optimal quality care. Acknowledging the practices of diverse cultures will allow professional nurses to provide a range of alternatives in services, for example, in providing the patient's dietary preferences or health care beliefs [11, 13].

 Communication barriers between nurses and their patients and families may arise because of cultural differences. It is important for nurses to recognize that caregiving within the family context may differ from their own interpretation and knowing how to integrate the family's health practices into the overall health care regimen will benefit both patient and family [14]. 

 Nursing curricula must provide a foundation for the development of cultural competence that allows for acquisition of knowledge, skills, and attitudes. In addition, an examination of culturally diverse concepts stimulates commitment to moral and ethical values, while developing an appreciation for diverse cultural heritages. Acquisition of culturally competent concepts promotes an awareness of contemporary world issues and can be learned through multiple teaching strategies, including didactic and experiential methods [15–18]. 

 In studies of cultural competence education in nursing, findings support that the addition of culturally competent content increases scores on culturally competence measures, as students gain experience during progression through the curricula [19–23]. A more recent review of the literature supports the findings of these studies [24]. Similarly, qualitative studies that examined cultural competence discovered emerging models for increasing cultural competence through education based on the patients' perspective, as well as that of nurse, and student nurses [25–29]. 

## 2. Purpose, Rationale, and Research Question of the Study

 The purpose of this study was to determine if the self-perception of cultural competence in baccalaureate nursing students as a result of their education and experiences increased during the nursing program. As the curriculum committee members were examining the existing nursing curriculum and planning a major revision, this study was instrumental in informing the faculty of the perceived cultural competence of beginning and graduating nursing students. Data generated by this study allowed nurse educators a better understanding of student perceptions of cultural competence and informed the curriculum committee of the cultural competence gained throughout the nursing program. The research question which framed this study was as follows: is there a higher perception of cultural competence among graduating nursing students as compared to beginning nursing students? 

 For the purpose of this study, culture was defined as beliefs and values of a particular group that are learned and shared and are generally transmitted between generations and influence thinking, decisions, and actions [30]. Cultural competence was theoretically defined as a process by which nurses strive to achieve the ability and availability to work within a cultural context of a patient, family, or community [31]. Cultural competence was operationally defined by a total score on the Cultural Competence Assessment (CCA) tool [20]. A beginning nursing student was defined as a baccalaureate nursing student in the first clinical semester of the nursing curriculum. A graduating nursing student was defined as a baccalaureate nursing student in the last clinical semester of the nursing curriculum.

## 3. Theoretical Frameworks

The major focus of transcultural nursing is to focus on the humanistic and scientific study of individuals from different cultures with consideration to ways in which nurses can assist those individuals meet their health and living needs [32]. Leininger's Transcultural Nursing Theory posits that caring serves to improve human conditions through behaviors, techniques, processes, and patterns. In addition, caring behaviors such as comfort, compassion, concern, interest, tenderness, touching, and trust are illuminated as key concepts to providing quality care through the lens of culture. Culture is determined by one's personal life and worldviews. Caring and culture are linked to one another, and nursing care should be aimed at preserving, maintaining, accommodating, negotiating, and restructuring care patterns as these relate to the individual's cultural perspectives [33]. 

 The 3-Dimensional Puzzle Model of Culturally Congruent Care was also chosen to guide this study. The four basic components of the cultural competence puzzle at the health care provider level include cultural diversity, cultural awareness, cultural sensitivity, and cultural competence [34]. The model is specifically operationalized using the CCA. This study tested quantitatively the fourth puzzle piece, cultural competence, using the CCA, as it measures perception to the actions taken in response to cultural diversity, cultural awareness, and cultural sensitivity. 

## 4. Methods and Procedures

The Institutional Review Board (IRB) approved the research study. Guidelines set forth by the Family Educational Rights and Privacy Act (FERPA) were followed to protect the privacy of student education records, and there was no identifying information collected. The design of this study was a comparative, descriptive design, testing for differences between beginning nursing students (*n* = 46) in the first clinical course and the last clinical course (*n* = 53) in a baccalaureate nursing program. Students were recruited during the first class meeting of the semester, and data were collected during the first week of classes using the CCA. The rationale for using a comparative design with data collection between two groups at one time instead of a longitudinal time frame allowed faculty to assess this concept in the current curriculum prior to a planned revision.

 The CCA is a 43- item Likert scale and was administered to gather data about individual self-perceptions regarding culture competence. The CCA had demonstrated in a previously reported study test-retest reliability (*r* = 0.85, *P* = .002; [20]). The CCA is made up of three different subscales, including the Cultural Awareness and Sensitivity Subscale (CAS) consisting of an 11-question Likert scale; the Cultural Competence Behavior Subscale (CCB) consisting of a 14-question Likert scale; and the Marlowe-Crown Social Desirability Scale consisting of 13 questions that were answered with either true or false by the student participants. The CAS is scored using a range of 1 to 7, with a higher score indicating a greater cultural awareness and sensitivity. The CCB is also scored using a range of 1 to 7, with a higher score indicative of more cultural competence behaviors being demonstrated. The Marlowe-Crown Social Desirability Scale was added to the instrument as a check/balance for honesty on the CCB subscale. This scale is scored using a range of 0 to 13, with a higher score indicating more need for approval, which indicates that respondents answered the questions according to what they believed to be socially acceptable [20]. 

 The data collected were analyzed using the independent *t*-test. This study measured perceived cultural competence as a total score upon which each groups' mean score was determined and subjected to independent *t* testing to determine differences between groups. The level of significance was set at *P* < .05. The data were analyzed using the Statistical Package for the Social Sciences (SPSS) 17.0 software.

## 5. Findings

There were a total of 99 nursing students, with an age range for the beginning nursing student participants of 19–35 years (*M* = 22.54) and an age range of the graduating nursing student participants of 21–65 years (*M* = 27.53). The average age of the graduating nursing students was statistically higher compared to the average age of the beginning nursing students (*t*[df = 97] = −3.617, *P* = .000). The racial/ethnic self-identification of the beginning nursing students included 24% Hispanic/Latino, 61% White/Caucasian, 4% Black/African American, and 11% Asian. The racial/ethnic self-identification of the graduating nursing students included 4% Hispanic/Latino, 72% White/Caucasian, 19% Black/African American, and 4% Asian. 

 The mean scores for the beginning and graduating nursing students are reported in Table 1. The graduating nursing students demonstrated a higher mean score for the CAS and the CCB (e.g., 6.018 and 4.590, resp.) as compared to the beginning nursing students (e.g., 5.695 and 3.453, resp.), indicating that the graduating nursing students have greater perceived cultural awareness and sensitivity and perceive that they demonstrate more culturally competent behaviors. However, it was noted that the beginning nursing students demonstrated a lower score on the Marlowe-Crown Social Desirability Scale as compared to the graduating nursing students (e.g., 7.00 and 7.75, resp.). This indicates that the beginning nursing students have a slightly less need for approval than do the graduating nursing students. Independent *t*-test findings demonstrated that perceptions of cultural diversity were significantly higher in the graduating nursing student group as compared with beginning nursing students (*t*[df = 97] = −3.233, *P* = .002). 

 One confounding variable encountered was age; the graduating nursing students' age was great, which was a statistically significant difference between groups. Therefore, a generalized linear model was conducted, using the CCA scores as the dependent variable and age as a predictor (*X* ^2^[df = 23] = 22.47, *P* = .49), which suggests that older age alone is not a sufficient variable for being culturally competent. Univariate analysis of variance on the dependent variable of the CCA scores with age held as the covariate was not statistically significant (*F* = 0.60[df = 1], *P* = .80). The amount of variance in the dependent variable that is shared with age is 0.1% (*R* ^2^ = .001). Reliability statistics were completed on the CCA for this sample, which resulted in Cronbach's Alpha = .823. 

## 6. Discussion

In examining the existing curriculum during a major revision, the concept of cultural competence was investigated for evidence of students' perception of their level of cultural competence. Extant research supports education, including a variety of teaching strategies in cultural diversity concepts, increases one's cultural competence. Study findings revealed a statistically significant difference in perceived level of cultural competence between the two groups, with graduating nursing students possessing a higher perceived level of cultural competence than the beginning nursing students. This indicates that graduating nursing students perceive that they have greater cultural awareness and sensitivity and have a greater understanding of what constitutes culturally competent behaviors. One assumption based on these results is that the role transition being experienced by the graduating nursing students may have influenced their answers. Oftentimes, there is a desire, on behalf of graduating nursing students, to be accepted in a Registered Nurse (RN) role rather than in a student role, and as such, graduating students may have answered the way they think an RN would have answered, as evidenced by their higher scores on the Marlowe-Crown Social Desirability Scale. 

 The findings of this study cannot be generalized beyond the geographical setting, as these were specific to the nursing population at this university. It is assumed that the findings of this study may not be similar to other nursing programs. All measures of cultural competence currently available are based on participant perceptions, which can be another limitation of the study. While the participants were chosen through convenience sampling, the entire student population of beginning and graduating students was invited to participate, and all who were present on the day of data collection did so. An additional limitation was that only two cohorts were examined in one measurement in time, whereas an ongoing analysis would provide stronger results.

## 7. Future Recommendations

There is an opportunity for nursing faculty at this university to use the information presented in this research study to develop additional culturally based student learning activities that will further increase the level of cultural competence in current and future baccalaureate nursing students. Specifically, based on the results of this study, additional activities have been added throughout the curriculum. It is recommended that all nursing courses address cultural competence in didactic activities as well as laboratory experiences, including simulation and clinical. It is recommended that a longitudinal study be utilized throughout all levels of baccalaureate nursing education to measure progress of cultural competence education as each student cohort progresses through the nursing program. 

Although the findings in this study are specific to the nursing population at one university, other universities can use the same assessment tool to gather information about the perceptions of cultural competence of their students in order to evaluate processes that are currently in place or to gather information that will assist them in the development of new teaching methodologies.

## Figures and Tables

**Table 1:** Subscale mean scores.

Student group	CAS subscale	CCB subscale	Marlowe-Crown Social Desirability Scale
Beginning student			
Mean	5.695	3.453	7.00
*N*	46	46	46
Standard deviation	4.544	1.210	2.477
Minimum score	4.454	0.000	1
Maximum score	6.545	6.000	13
Graduating student			
Mean	6.018	4.590	7.75
*N*	53	53	53
Standard deviation	5.295	1.132	2.336
Minimum score	4.727	2.142	3
Maximum score	7.000	6.785	12
